# Improved Olfactory Deposition of Theophylline Using a Nanotech Soft Mist Nozzle Chip

**DOI:** 10.3390/pharmaceutics16010002

**Published:** 2023-12-19

**Authors:** Madeline X. Zhang, Frank Verhoeven, Pieter Ravensbergen, Stefan Kooij, Rick Geoffrion, Daniel Bonn, Cees J. M. van Rijn

**Affiliations:** 1 Van der Waals-Zeeman Institute, Institute of Physics, University of Amsterdam, 1098 XH Amsterdam, The Netherlands; s.a.kooij@uva.nl (S.K.); d.bonn@uva.nl (D.B.); c.j.m.vanrijn@uva.nl (C.J.M.v.R.); 2 Medspray B.V., 7521 PV Enschede, The Netherlands; frank@medspray.com (F.V.); pieter@medspray.com (P.R.); 3 Cyrano Therapeutics Inc., Delray Beach, FL 33445, USA; rick@cyranotherapeutics.com

**Keywords:** aerosol, nebulizer, olfactory region, intranasal delivery, soft mist, nanotechnology

## Abstract

Currently, nasal administration of active pharmaceutical ingredients is most commonly performed using swirl-nozzle-based pump devices or pressurized syringes. However, they lead to limited deposition in the more active regions of the nasal cavity, especially the olfactory region, which is crucial for nose-to-brain drug delivery. This research proposes to improve deposition in the olfactory region by replacing the swirl nozzle with a nanoengineered nozzle chip containing micrometer-sized holes, which generates smaller droplets of 10–50 μm travelling at a lower plume velocity. Two nanotech nozzle chips with different hole sizes were tested at different inhalation flow rates to examine the deposition patterns of theophylline, a hyposmia treatment formulation, using a nasal cavity model. A user study was also conducted and showed that the patient instructions influenced the inhalation flow rate characteristics. Targeted flow rates of between 0 and 25 L/min were used for the in vitro deposition study, yielding 21.5–31.5% olfactory coverage. In contrast, the traditional swirl nozzle provided only 10.8% coverage at a similar flow rate. This work highlights the potential of the nanotech soft mist nozzle for improved intranasal drug delivery, particularly to the olfactory region.

## 1. Introduction

The nasal tract is considered an efficient pathway for the uptake of biopharmaceuticals and small molecules, as the nasal cavity is highly vascularized and has a large surface area. In the nasal mucosae, a variety of antigen-presenting cells are present, such as macrophages and dendritic cells, which continuously scan their environment for uninvited antigens [1,2]. Over the years, various nasal drug delivery devices, such as vibrating mesh nebulizers [3], propellant pressurized sprays, aqueous spray pumps, and dry powders have been used. However, aqueous spray pumps are now dominant [4,5]. This is surprising as experimental studies have shown that many spray pump devices deposit a significant amount of the drug in the nasal valve and vestibule, which are considered less active regions of the nasal cavity [6,7,8]. This deposition in the nasal valve and vestibule is likely due to inertial impaction since most spray pumps are designed to release a large proportion of aerosol particles substantially larger than 50 μm, which exit the devices at a high speed [9]. Several studies have suggested that nasal nebulization with much smaller droplets (<50 μm) is a more effective method of delivering topical medication beyond the nasal valve region than aqueous spray pumps [4,5,7,10]. This enhanced penetration is attributed to the fact that nebulizers are designed to generate small, slow-moving particles that traverse the nasal cavity at a resting breathing rate, thereby minimizing the inertial impact on the nasal valve and vestibule [5,7]. In addition to the delivery device, a combination of other factors can also contribute to the efficacy of the intranasal formulations. These include the drug formulation characteristics, the site of deposition, and the patient instructions for using the delivery device [11]. 

The olfactory region, located in the upper part of the nasal cavity, is responsible for the sense of smell, which became especially clear to patients with infection-related hyposmia during the SARS-CoV-2 pandemic. Theophylline, a natural alkaloid derivative of xanthine isolated from Camellia sinensis and Coffee arabica plants, has been shown to improve lost taste and smell when administered intranasally [12]. Theophylline appears to inhibit phosphodiesterase (PDE) and prostaglandin production, regulate calcium flux and intracellular calcium distribution, and antagonize adenosine. By inhibiting PDE, theophylline may inhibit the conversion of cAMP to AMP, increasing its concentration in the mucosal microenvironment, and thereby augmenting olfactory transduction stimulation [13]. Over time, the receptor/sensory neurons may repopulate and become stimulated, allowing the brain to process odorants. Furthermore, the olfactory region is also known to provide a pathway to deliver formulations directly to the brain through the blood–brain barrier [14], making it a unique and important region for intranasal delivery. However, normally, with current atomization techniques, only <5–10% of the formulation that is administered intranasally deposits on the olfactory mucosa [15], posing a significant challenge in achieving sufficient drug efficacy.

In this research, we performed an in vitro deposition study to investigate the extent of theophylline deposition in the olfactory region using a novel nanotech nozzle chip in combination with a simple spray pump. The nanotech nozzle chip consists of a silicon chip with micrometer-sized pores, which emits a slow-moving spray cloud upon spraying with smaller droplets and a narrower droplet size distribution than the conventional swirl nozzle [9]. It is made with semi-conductor technology, which provides several advantages for production in addition to the potential improvements in the nasal deposition investigated in this work. First, well-defined pore sizes can be maintained during the manufacturing of the nozzle chip. Compared to the effect of user instruction and user nasal dimensional variations on the deposition and efficacy, the small difference in pore size due to potential production inaccuracies is expected to have a negligible effect. Moreover, the production of the nanotech nozzle chip is scalable, as semi-conductor technology enables lower costs at larger production volumes, making the technology affordable for both pharmaceutical and OTC applications. 

In addition to the deposition study, a user study was performed to measure the effect of user instructions by examining the inhalation pattern of volunteers when given different inhalation instructions. These inhalation patterns were used as inputs for the deposition study. 

## 2. Materials and Methods

### 2.1. User Study

Inhalation pattern measurements were performed to identify the flow rate and the duration of inhalation. The volunteers were instructed to inhale from a nasal inhalation device consisting of a disposable nasal adaptor and a flowmeter (Sensirion SFM3019, Stäfa, Switzerland) under several different inhalation instructions. The small opening of the device was placed inside one nostril to simulate inhalation from a nasal spray pump, while the second nostril was blocked.

A total of 32 volunteers were sampled, mainly staff and students from the University of Amsterdam. The volunteers were between 21 and 67 years old, with a mean age of 35 years, and 37.5% were female (12 out of the 32). Five versions of user instructions were defined and are listed in Table 1. The instructions were given in the order described, and inhalation measurements were taken after reading each instruction to the volunteer. Volunteers were not specifically instructed to exhale before inhalation. The flow rate time series were recorded at a sampling rate of 100 Hz. 

### 2.2. In Vitro Deposition Study 

**Figure 1:**
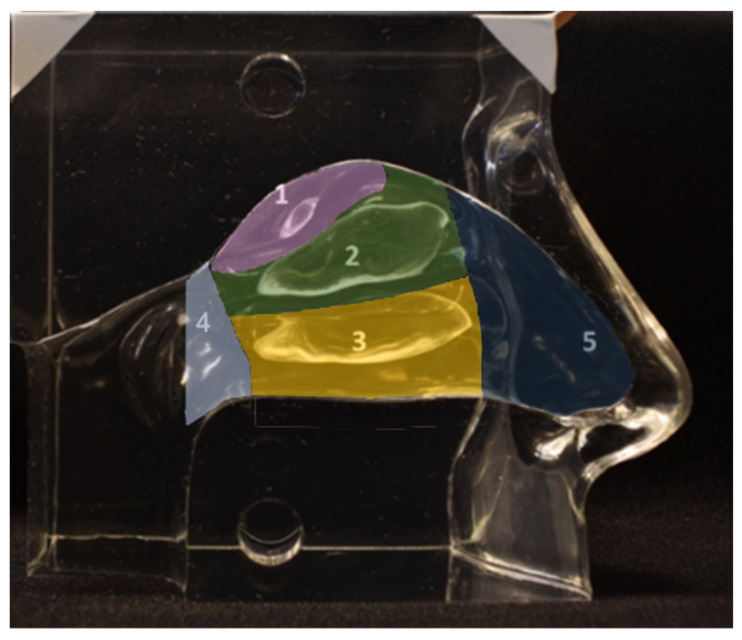
**The** image analysis mask with the intranasal regions of interest projected onto the nasal cast, showing (1) the olfactory region; (2) the middle turbinate; (3) the inferior turbinate; (4) the nasopharynx; (5) the vestibule. The mask was used for the photo analysis to determine the coverage in each region.

Deposition experiments were performed to determine the deposition characteristics of the nanotech nebulization chip under different conditions. The experimental procedures of the deposition study were adapted from D’Angelo et al. [9]. A transparent silicone nasal cavity model (Model LM-005 Koken Ltd., Tokyo, Japan) was used to simulate the nasal cavity. Figure 1 shows the nasal cast and corresponding intranasal regions used for analysis. During spraying, only half of the model, containing one nostril/nasal cavity with the septum attached, was used. 

**Figure 2:**
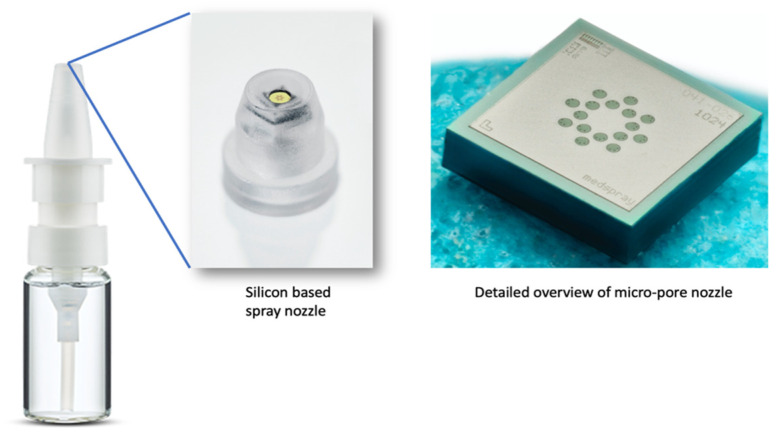
The nanotech nebulization device consisting of a nasal spray pump (Aero Pump GmbH, Hochheim, Germany) and an integrated silicon-based nanotech (soft mist) nozzle chip (Medspray, Enschede, The Netherlands) (image obtained from “Fluorescence-enabled evaluation of nasal tract deposition and coverage of pharmaceutical formulations in a silicone nasal cast using an innovative spray device” by D’Angelo et al. [9] licensed under CC BY-NC-ND 4.0 DEED).

**Figure 3:**
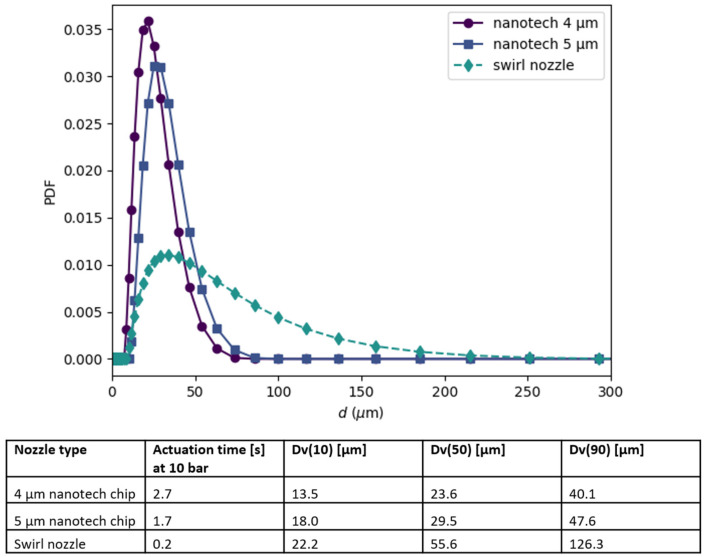
Top: the droplet size distribution (probability density function) produced by the two nanotech nozzle chips with pore sizes of 4 µm and 5 µm and a nasal swirl nozzle measured by a Malvern Spraytec laser diffraction system. Bottom: the actuation times and the droplet size volume percentiles for the three nozzles.

A 45 µL nasal spray pump in combination with two nanotech nozzle chips from Medspray (Enschede, The Netherlands) with different hole sizes, as shown in Figure 2, was used in the deposition experiments. Each nanotech nozzle had a silicon chip containing either 48 pores of 4 µm in diameter or 48 pores of 5 µm in diameter, both of which generated a slow-moving soft mist with a fixed spray plume angle of 20°. The nozzle chips with the 4 µm and the 5 µm pores created droplets with a mean diameter (Dv50) of 23.6 µm and 29.5 µm and delivered the full dose of the aerosol in approximately 2.7 s and 1.7 s, respectively. The results of the droplet size measurements and the full-dose delivery times are shown in Figure 3. The spray pump was actuated inside the nostril at an angle of 45–60°, with respect to the palate, and at an insertion depth of 10 mm, depositing a mixture of theophylline and calcein, a fluorescent dye, inside the nasal cast. 

To prepare the theophylline–calcein mixture used for spraying, a calcein stock solution was first created. The formulation of the calcein stock solution was adapted from D’Angelo et al. [9]. Calcein powder was solubilized in 80 µL of 1M NaOH, to which 25% (*w*/*v*) glycerol and 0.9% saline (*v*/*v*) were added to obtain a calcein concentration of 40 mg/mL. The NaOH was used to increase the pH of the stock solution to a value of 10, as calcein can only be solubilized at high pH values, and glycerol was added to increase the viscosity of the solution to prevent dripping in the nasal cast after spraying. Theophylline was then added to the stock solution as a model drug until a final calcein concentration of 1mg/mL was obtained. The formulation composition was selected to preserve fluorescence over at least 15 min, which is a sufficient time to image the nasal cast after deposition. In all the experiments, the spray pump was actuated three times, resulting in a total of 135 µL of the solution nebulized per experiment. 

First, for comparing the two nanotech nozzle chips, deposition under a fixed flow was studied. To do so, a constant simulated inhalation flow of 15 L/min (as measured in the nostril) was maintained in the nasal cavity during spraying using a Copley LCP5 vacuum pump (Copley Scientific, Nottingham, UK) and a TSI 4040 flowmeter (TSI Incorporated, Shoreview United States, Shoreview, MN, USA). Measurements at the same flow rate were also performed for a nasal swirl nozzle for comparison with the nanotech nozzle chips.

Next, deposition experiments were performed at typical flow rates as found in the user study to investigate the effect of the flow rate on the deposition pattern.

Before and after spraying, images of the nasal cast were acquired using a Velleman^®^ UV Lamp (ZLUVB, Velleman NV, Gavere, Belgium) as a source of UV rays with a wavelength of 366 nm and a digital camera (Nikon D3400, Nikon, Tokyo, Japan) set at an exposure time of 1/5 s, an f-stop of 5.6, a focal distance of 18 mm, and an ISO number of 3200. To ensure standardized photographic conditions, all images were acquired inside a black box, and the camera was fixed at a distance of 15 cm from the cast. Before each image acquisition, the septum was removed. The images were analyzed with the software ImageJ 1.53k (U.S. National Institute of Health, Bethesda, MD, USA), where a 2D mapping of the deposition in the 3D model could be determined. The cast area was divided into fixed regions of interest (ROIs) for all of the images analyzed, as shown in Figure 1. The intensity range was kept fixed between 11 and 256. Each experiment was repeated ten times.

## 3. Results

### 3.1. Effect of the User Instruction on the Inhalation Flow Characteristics

**Figure 4:**
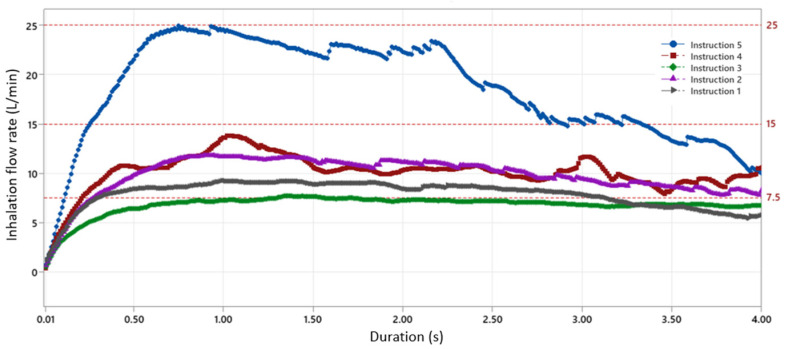
The average inhalation flow rate for different instructions. The flow rates used for the deposition studies are shown as red dotted lines (7.5, 15, 25 L/min). For each duration, averaging was performed over all the volunteers, excluding data from the volunteers who did not reach that duration.

**Figure 5:**
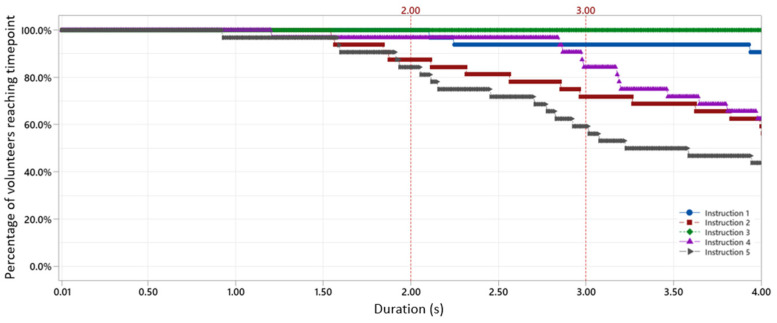
The percentage of the volunteers reaching a certain inhalation duration. The red dotted line at 2 s indicates the minimum time needed to deposit the complete aerosol dose.

The outputs of the user study are the inhalation duration, the mean inhalation velocity, and the mean peak inhalation velocity for each of the instructions, as tabulated in Table S1. For the nanotech nasal spray to deliver the full dose, it is essential that the duration of the inhalation is sufficiently long. Using the 4 µm nanotech nebulizer as a reference, this is approximately 2 s as shown in Figure 3. Figure 4 shows the inhalation flow during the first 4 s of the inhalation maneuver averaged over all the volunteers. The percentage of the volunteers that reached a certain inhalation duration is shown in Figure 5. While all versions of the instructions achieved a sufficient inhalation time (i.e., 2 s) to deliver the entire dose for 80% of the volunteers, only instruction 3 achieved it for 100% of the volunteers. For instruction 4, the inhalation pattern consists of several short sniffs for some volunteers, resulting in a less constant flow, which is not ideal for delivering a longer-lasting soft mist nasal spray. Instruction 5 resulted in a large variation in the inhalation velocities between the volunteers. Overall, instructions 1 and 3 resulted in the most stable inhalation curve with a small variance and sufficiently long inhalation times. Based on Figure 4, we chose four flow rates: 0, 7.5, 15, and 25 L/min for the in vitro deposition studies, as the user study suggests that they are representative of in vivo conditions.

### 3.2. Effect of the Nozzle Parameters on the Deposition Pattern at a Fixed Inhalation Flow Rate

**Figure 6:**
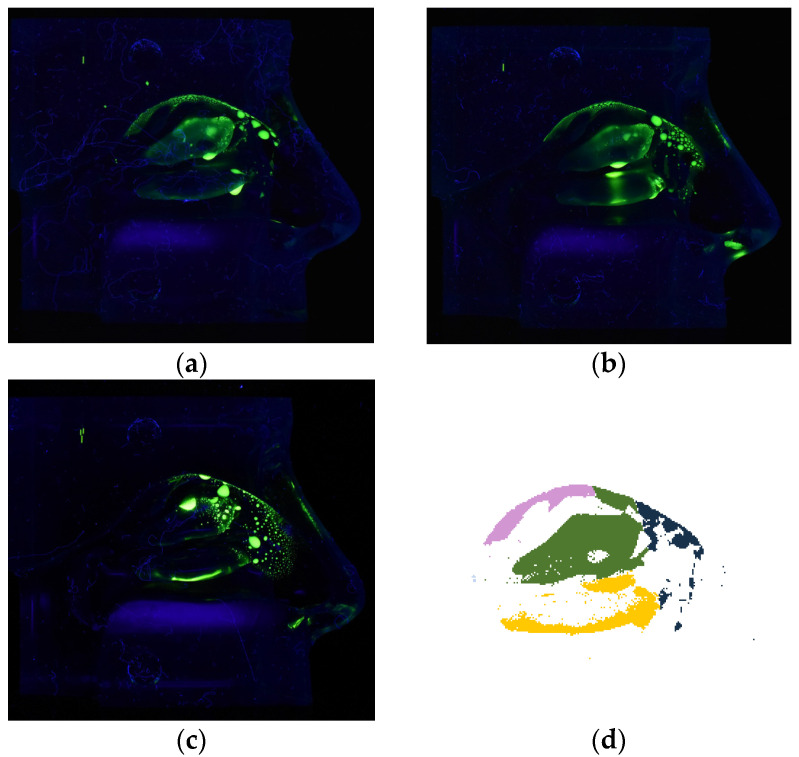
Fluorescence images of nasal cast coverage with calcein-stained theophylline solution deposited at a flow rate of 15 L/min: (**a**) 4 µm nanotech nozzle chip, (**b**) 5 µm nanotech nozzle chip, (**c**) swirl-nozzle. (**d**) An example photo-analysis of (**a**) with different regions colored (pink: olfactory region, green: middle turbinate, dark blue: nostril, yellow: inferior turbinate). Note that the nasopharynx is not visible, as this region is covered only for <0.6% in this scenario.

For the deposition experiments conducted at a fixed inhalation flow rate of 15 L/min, Figure 6 shows the image comparison between the different nebulization devices in terms of the deposition pattern. It also demonstrates how an image is divided into regions, for which the covered area relative to the whole surface area of that region is calculated, as tabulated in Table S2 and summarized in Table 2. Although the deposition coverage in the nostril is similar for both types of devices, there is more coverage for the nanotech nozzle chips than for the swirl nozzle in the middle turbinate, the inferior turbinate, and especially in the olfactory region. The image also shows that the swirl nozzle leads to deposition mainly in the anterior region of the nasal cavity, and the coverage in the middle and inferior turbinate is very non-uniform. No significant difference is observed between the two nanotech nozzle chips with different size holes, reflecting the relatively small differences in the plume velocity, the spray pattern, and the droplet sizes (see Figure 3).

### 3.3. Effect of the Inhalation Flow Rate on the Deposition Pattern

**Figure 7:**
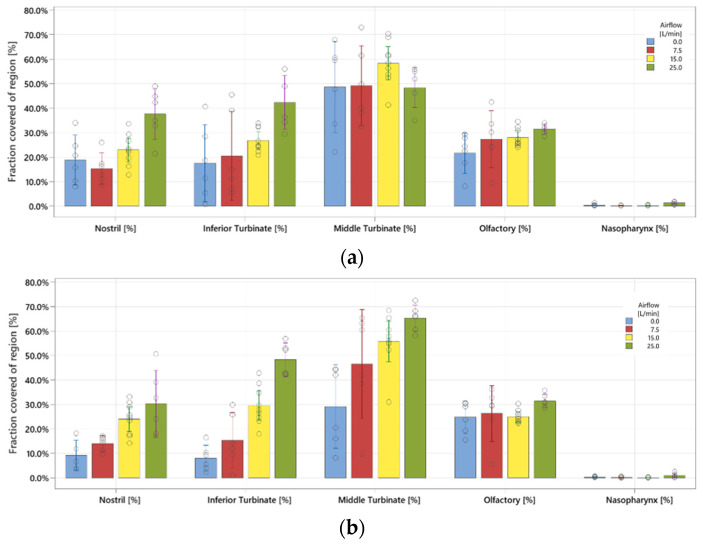
The deposition in different regions with the (**a**) 4 µm and (**b**) 5 µm nanotech nozzle chips at various flow rates.

**Figure 8:**
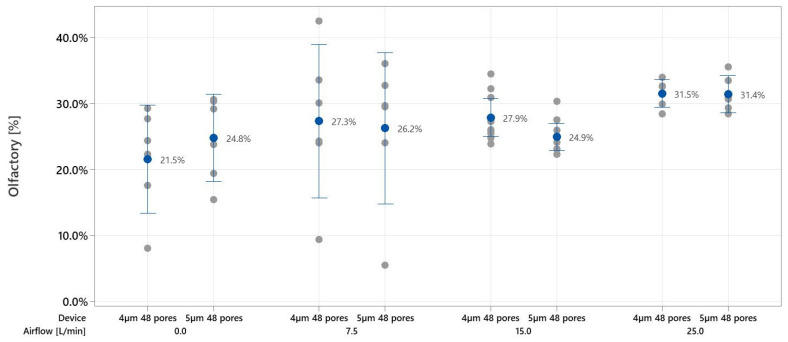
The olfactory region deposition for the two nanotech nozzle chips at various flow rates.

For the experiments investigating the effect of the inhalation flow rates on the deposition in the nasal cavity, Figure 7a,b show the relative area coverages of each region for the four inhalation flow rates, using the 4 µm and 5 µm nanotech nozzle chips, respectively. The values are tabulated in Table S3. In two regions (in the case of the 4 µm nozzle) and all four regions (5 µm nozzle), the deposition increases slightly with increasing inhalation velocity. Overall, the deposition for the 5 µm nozzle chip has a stronger correlation with the flow rate. Deposition in the olfactory region appears least affected by the inhalation flow rate when compared to the other regions and only very slightly increases with the increasing flow rate. However, at lower inhalation velocities the variance in the olfactory coverage increases. Figure 8 compares the olfactory coverage for the two nanotech nozzle chips and the four flow rates. No significant differences are observed between the nozzles at each inhalation velocity. Both nanotech nozzle chips achieved an olfactory coverage of more than 20% when holding breath and more than 30% for a flow rate of 25 L/min.

## 4. Discussion

The nasal cavity coverage and deposition characteristics are contingent upon the droplet size and the droplet velocity. Figure 3 shows that the swirl nozzle generates brief bursts of droplets with a wide size distribution, of which a large proportion is above 50 μm in diameter. These relatively large droplets ejected at a high velocity from the swirl nozzle follow a straight ballistic path at speeds exceeding 10 m per second [9]. Consequently, the majority of these large droplets are deposited in the front of the nasal cavity (Figure 6c), as the inertially-driven swirl nozzle droplets cannot navigate curved trajectories to reach the back of the nasal cavity. Furthermore, the interplay between the surface tension and inertial forces precludes the reduction in droplet size using a swirl nozzle. On the other hand, electric vibrating mesh nebulizers typically produce very small droplets around 5 μm [3], which are able to follow the airflow patterns more easily. However, these small droplets also more easily pass through the whole nasal cavity and continue down into the lower respiratory tract.

The nanotech nozzle device produces medium-sized droplets of around 10–50 μm (Figure 3). At this droplet size range, the droplets are likely to be in a favorable translational regime. As such, they are able to avoid ballistic impact in the anterior parts of the nasal cavity and follow the curved trajectory at a slower velocity (<1 m/s) [9], thus depositing more in the middle regions. As a result, the nanotech nozzle chip demonstrates superior deposition in the turbinate regions, including the olfactory region (Figure 8), and low passage to the nasopharyngeal region. Hence, our study showed that the slow-moving droplets of around 10–50 μm are suitable for olfactory deposition, a result that corroborates previous studies [6,16]. 

In the deposition study, the effect of droplet size was further tested using two different-sized nozzle chips. In both cases, the spraying duration was significantly longer than a classical swirl nozzle nasal pump (Figure 3), resulting in a lower plume velocity and a mean droplet diameter of around 25 µm and 30 µm for the 4 μm and 5 μm nanotech nozzle chips, respectively. The similar droplet sizes explain why the deposition results for the two nanotech nozzle chips are not significantly different for the same simulated inhalation flow.

Next, the user study shows that the different instructions lead to different flow rate patterns and suggests that instructions 1 and 3 provide the most constant flow rate over time, while instruction 5 gives the highest flow rate. Considering the effect of the inhalation flowrate on the deposition in the different regions inside the nasal cavity, a clear trend is visible for the 5 µm nozzle configuration (Figure 7b), which indicates that a significantly higher surface coverage can be obtained in the “nostril”, “inferior turbinate”, and “middle turbinate” regions at a higher inhalation velocity. This is likely because the inertial effect is stronger at higher flow rates, leading to more droplets impacting the mucosal surface in these relatively frontal regions. Therefore, in scenarios where the olfactory region is not the target delivery region, a more forceful inhalation at a higher mean inhalation flow rate between 15 and 25 L/min will give a significantly higher surface coverage in the other nasal regions. In those scenarios, provided that patients are able to inhale for long enough, higher inhalation velocities through instructions such as “inhale firmly for at least 2 s” will likely give the best results, although it is also likely to result in more variability from patient to patient. 

On the other hand, deposition in the olfactory region is less affected by the inhalation flow rate. This demonstrates the robustness of the nanotech nozzle for olfactory delivery, as high area coverage (>20%) of the olfactory region can be achieved for any flow rate within the range of 0–25 L/min, making it suitable for a range of patients with different breathing patterns, provided that the inhalation time is sufficient. Since the deposition study only examined scenarios with constant flow rates, the effect of the time-varying flow rate on deposition requires future investigations but is expected to be small for realistic breathing patterns such as those obtained from the user study.

## 5. Conclusions

In this study, the suitability of a novel nanotech soft mist nasal spray device for the nebulization of theophylline towards the olfactory region was demonstrated. The effects of user instructions on the flow rate and subsequent deposition pattern were investigated through a user study and a deposition study. The nanotech nozzle chips achieved 21.5–31.5% coverage in the olfactory region for flow rates between 0 and 25 L/min, which is significantly higher than what is obtainable with conventional swirl nozzles. The two nanotech nozzle chips with different pore sizes did not differ significantly in terms of nasal coverage, as the droplet size distributions of the two chips are similar. The flow rate did not affect the olfactory deposition significantly within this range of flow rates tested. In future studies, the relative importance of the droplet size distribution and the spray velocity may be determined by manufacturing and testing nanotech nozzle chips with different numbers of pores, more pore sizes, and incorporating multiple pore sizes on the same chip. 

In addition to hyposmia treatment with theophylline, more intranasal applications may benefit from the use of this nanotech nozzle chip technology, such as selective drug administration to the olfactory region for effective nose-to-brain drug delivery of biologics [17], oxytocin [18], polypeptides [19], and short interfering RNA for gene silencing [20], as the olfactory region can be used for crossing the blood–brain barrier. Furthermore, intranasal vaccination strategies might benefit from a more uniform formulation coverage of the mucosal tissue with the nanotech nozzle chip [21]. The pore size and the configuration of the nozzle chip may be customized for different applications to optimize the delivery.

## Figures and Tables

**Table 1:** The instructions used in the user study.

#	Instruction
1	“Inhale gently through the nose, do not sniff.”
2	“Inhale normally through the nose.”
3	“Inhale slowly through the nose.”
4	“Inhale with the idea that you would like to smell something that is difficult to smell.”
5	“Inhale firmly through the nose.”

**Table 2:** An overview of the nasal coverage data for the two different nanotech nozzle chips (4 µm and 5 µm pore size) and a swirl nozzle all measured at a 15 L/min airflow. The percentages are based on the relative coverage of the region, compared to the total surface area of the region.

Device Info	Region Coverage Percentages					
Nozzle Type	Total Nasal Cast [%]	Nostril [%]	Inferior Turbinate [%]	Middle Turbinate [%]	Olfactory [%]	Nasopharynx [%]
Nanotech chip 4 µm × 48 pores	30.5% (±2.9%)	22.9% (±6.4%)	26.8% (±4.8%)	58.3% (±8.8%)	27.9% (±3.7%)	0.2% (±0.2%)
Nanotech chip 5 µm × 48 pores	29.6% (±4.9%)	23.1% (±6.5%)	27.8% (±7.9%)	54.1% (±10.6%)	24.8% (±2.6%)	0.1% (±0.1%)
Swirl nozzle	19.7% (±1.2%)	21.2% (±0.4%)	13.9% (±4.0%)	37.0% (±1.1%)	10.8% (±1.4%)	1.3% (±1.0%)

## Data Availability

The data presented in this study are available in the main text of this article or Supplementary Materials.

## Supplementary Materials

The following supporting information can be downloaded at: https://www.mdpi.com/article/10.3390/pharmaceutics16010002/s1, Table S1: Summary of flow measurements under the different inhalation instructions listed in Table 1; Table S2: Summary of flow measurements under the different inhalation instructions listed in Table 1; Table S3: Summarized nasal coverage data for the two nanotech nozzle chips (4 µm and 5 µm) and four airflows (0, 7.5, 15, 25 L/min).
