# Supplementary material for: Improved Olfactory Deposition of Theophylline Using a Nanotech Soft Mist Nozzle Chip
Source: Pharmaceutics. 2023 Dec 19;16(1):2. doi: 10.3390/pharmaceutics16010002 (PMC10821129; doi:10.3390/pharmaceutics16010002)
Supplement: Supplementary file 1 [file pharmaceutics-16-00002-s001.zip › pharmaceutics-2739976-supplementary.pdf]

Supplementary materials

Table S1 | Summary of flow measurements under the different inhalation instructions listed in Table 1.

Instruction		1	2	3	4	5
**Mean inhalation flow (L/min)**	mean	7.84	9.50	6.36	10.83	18.54
	SD	4.11	4.00	3.41	5.04	11.48
	min	3.02	4.17	2.10	3.58	4.93
	max	16.88	19.10	14.47	23.41	62.44
**Peak inhalation flow (L/min)**	mean	11.54	13.98	9.51	18.29	28.94
	SD	5.28	4.98	3.80	8.97	17.73
	min	4.90	5.53	3.60	5.63	8.07
	max	21.33	24.13	18.43	45.47	98.57
**Mean flow during first 2 seconds of inhalation (L/min)**	mean	8.41	10.15	6.43	10.66	19.97
	SD	4.49	4.06	3.30	4.83	13.01

Table S2 | Summary of flow measurements under the different inhalation instructions listed in Table 1. Individual data-points of in total 20 separate measurements of two different nanotech nozzle chips (4µm and 5µm) measured at 15 L/min airflow. Percentages are based on relative coverage of each region relative to the total surface area of that region.

Nanotech nozzle	Total surface coverage [%]	Nostril coverage [%]	Inferior turbinate coverage [%]	Middle turbinate coverage [%]	Olfactory coverage [%]	Nasopharynx coverage [%]
4µm	31.3%	33.6%	33.7%	41.3%	24.8%	0.3%
4µm	28.1%	22.8%	24.2%	52.4%	26.0%	0.2%
4µm	33.8%	26.6%	32.5%	61.8%	25.8%	0.1%
4µm	26.7%	16.3%	22.2%	56.2%	27.2%	0.1%
4µm	27.3%	22.2%	20.7%	53.7%	25.5%	0.0%
4µm	35.1%	23.4%	32.2%	68.9%	32.2%	0.2%
4µm	32.6%	29.3%	24.5%	61.2%	30.9%	0.0%
4µm	30.5%	12.7%	24.8%	70.2%	34.4%	0.6%
4µm	29.2%	19.5%	26.6%	58.5%	23.9%	0.1%
5µm	26.2%	17.9%	23.2%	52.3%	24.1%	0.3%
5µm	32.2%	23.5%	34.8%	55.9%	25.0%	0.1%
5µm	30.4%	24.6%	26.5%	54.9%	30.3%	0.1%
5µm	28.2%	17.1%	24.6%	60.5%	23.1%	0.0%
5µm	24.0%	33.1%	17.9%	31.0%	22.3%	0.1%
5µm	27.6%	14.1%	29.6%	55.1%	23.0%	0.1%
5µm	31.8%	29.0%	27.6%	57.7%	23.0%	0.2%
5µm	36.3%	25.6%	38.7%	65.3%	27.5%	0.1%
5µm	39.4%	30.8%	42.8%	68.4%	25.9%	0.1%

Table S3 | Summarized nasal coverage data for the two nanotech nozzle chips (4µm and 5µm) and four airflows (0, 7.5, 15, 25 L/min). Percentages indicate the surface coverage of the olfactory region relative to the total surface area of that region.

Measurement conditions		Region coverage percentages					
**Nozzle chip**	**Airflow [L/min]**	**Total Nasal cast [%]**	**Nostril [%]**	**Inferior turbinate [%]**	**Middle turbinate [%]**	**Olfactory [%]**	**Nasopharynx [%]**
4µm x 48 pores	0	23.8% (SD 10.8%)	18.8% (SD 9.6%)	17.5% (SD 15.0%)	48.6% (SD 17.7%)	21.5% (SD 7.8%)	0.4% (SD 0.4%)
5µm x 48 pores	0	15.6% (SD 2.3%	8.7% (SD 6.2%)	9.1% (SD 4.9%)	33.4% (SD 13.9%)	26.7% (SD 4.9%)	0.4% (SD 0.1%)
4µm x 48 pores	7.5	24.4% (SD 10.0%)	15.3% (SD 6.2%)	20.5% (SD 17.3%)	49.1% (SD 15.5%)	27.3% (SD 11.1%)	0.1% (SD 0.2%)
5µm x 48 pores	7.5	21.9% (SD 8.7%)	14.1% (SD 3.0%)	15.4% (SD 10.7%)	46.5% (SD 21.2%)	26.2% (SD 10.9%)	0.2% (SD 0.2%)
4µm x 48 pores	15	30.5% (SD 2.9%)	22.9% (SD 6.4%)	26.8% (SD 4.8%)	58.3% (SD 8.8%)	27.9% (SD 3.7%)	0.2% (SD 0.2%)
5µm x 48 pores	15	29.6% (SD 4.9%)	23.1% (SD 6.5%)	27.8% (SD 7.9%)	54.1% (SD 10.6%)	24.8% (SD 2.6%)	0.1% (SD 0.1%)
4µm x 48 pores	25	37.5% (SD 5.4%)	37.5% (SD 10.0%)	42.3% (SD 10.4%)	48.3% (SD 7.8%)	31.5% (SD 2.0%)	1.3% (SD 0.6%)
5µm x 48 pores	25	41.0% (SD 5.8%)	30.3% (SD 13.1%)	48.2% (SD 6.5%)	65.2% (SD 5.2%)	31.4% (SD 2.7%)	1.0% (SD 0.8%)
